# Enhanced Gel Properties of Duck Myofibrillar Protein by Plasma-Activated Water: Through Mild Structure Modifications

**DOI:** 10.3390/foods12040877

**Published:** 2023-02-18

**Authors:** Wei Rao, M. S. Roopesh, Daodong Pan, Lihui Du

**Affiliations:** 1 State Key Laboratory for Managing Biotic and Chemical Threats to the Quality and Safety of Agro-Products, Ningbo 315211, China; 2 Key Laboratory of Animal Protein Food Processing Technology of Zhejiang Province, College of Food and Pharmaceutical Sciences, Ningbo University, Ningbo 315800, China; 3 Department of Agricultural, Food and Nutritional Science, University of Alberta, Edmonton, AB T6G 2P5, Canada

**Keywords:** plasma, gel, oxidation, protein conformation

## Abstract

This study assessed the gel properties and conformational changes of duck myofibrillar protein (DMP) affected by plasma-activated water (PAW) generated at various discharge times (0 s, 10 s, 20 s, 30 s, and 40 s). With the treatment of PAW-20 s, the gel strength and water-holding capacity (WHC) of DMP gels were significantly increased when compared to the control group. Throughout the heating process, dynamic rheology revealed that the PAW-treated DMP had a higher storage modulus than the control. The hydrophobic interactions between protein molecules were significantly improved by PAW, resulting in a more ordered and homogeneous gel microstructure. The increased sulfhydryl and carbonyl content in DMP indicated a higher degree of protein oxidation with PAW treatment. Additionally, the circular dichroism spectroscopy demonstrated that PAW induced α-helix and β-turn transformed to β-sheet in DMP. Surface hydrophobicity, fluorescence spectroscopy, and UV absorption spectroscopy suggested that PAW altered DMP’s tertiary structure, although the electrophoretic pattern indicated that the primary structure of DMP was mostly unaffected. These results suggest that PAW can improve the gel properties of DMP through mild alteration in its conformation.

## 1. Introduction

More than 4.5 million tons of duck are consumed worldwide each year, and the market demand for products made from duck meat is continually rising [1]. Asian countries account for more than 80% of the world’s total duck meat production, with China dominating more than half of duck meat production [1]. Duck meat is high in nutritional value, with great content of essential amino acids and fatty acids. The methionine content in duck legs is about 10 g/100 g protein, which is much higher than chicken [2]. C18:2 (13–23%) and C20:4 (8–19%) are the main fatty acids in duck legs, and their degradation products are important in processing [2]. The edible portion of duck meat contains more than 16% protein, and the essential amino acid index is as high as 86.02 [3]. Most of the fatty acids in duck meat are unsaturated fatty acids with a low melting point, which are easily digested and absorbed by the human body [4]. However, due to the relative backwardness of the waterfowl industry and insufficient deep processing of duck meat, much duck meat cannot be effectively developed and utilized, resulting in the waste of high-quality protein. Traditional duck products such as Beijing roast duck and Nanjing salted duck have gained popularity in many countries, but consumers are seeking greater diversity in taste and product offerings [4]. As a result, the development of new duck meat products is critical for maximizing the value of waterfowl protein resources and the expansion of the duck meat market scale.

Compared to the other types of meat products, the inferior functional properties of the protein in duck meat are the main limiting factor that restricts the development of duck meat products, such as lower emulsifying properties, lower-grade of gelling characteristics, etc., resulting in rough taste and poor palatability of duck meat products, and therefore decreased the acceptability of consumers [5,6]. The most important structural and functional protein in muscle is myofibrillar protein (MP), and its gelling capacity plays an important role in the texture and sensory properties of meat products [7,8]. Therefore, improving the gel properties of duck myofibrillar protein (DMP) would make a great contribution to the development, utilization, and quality improvement of duck meat products. Currently, there is limited research on methods to improve the gel properties of duck meat products. Despite this, there are some challenges that need to be addressed, such as low efficiency, high cost, safety issues, the risk of chemical residue, and the potential to negatively affect flavor and texture [4,9,10].

Plasma, which is comprised of electrons, positive ions, neutral particles, and free radicals, has captured the attention of researchers. This technology boasts high efficiency, leaves no residue, and is cost-effective. Numerous studies have documented its use in food processing, including the inactivation of microorganisms in food, the removal of pesticide residues from fruits and vegetables, and the degradation of mycotoxins in grain [11,12,13]. Previous studies have shown that plasma can change the structure of proteins to affect their functions [14,15]. Ekezie et al. [16] found that after treating shrimp MP with atmospheric pressure plasma jets, the protein’s secondary and tertiary structures changed and the hydrophobic groups were exposed. Luo et al. [17] found that the surface hydrophobicity and emulsification of pork MP were significantly increased after treatment with dielectric barrier discharge plasma. However, direct plasma treatment (direct contact between food and plasma) is characterized by uneven food contact, food overheating, and unstable effect [18]. Plasma-activated water (PAW) is a form of plasma existence with water as the medium. A large number of reactive oxygen species (ROS) and reactive nitrogen (RNS) contained in PAW can react with macromolecules in food, and PAW has shown the advantages of treating food evenly, avoiding excessive oxidation, etc. [19]. So far, the influence of PAW on the gel properties of DMP and its regulatory mechanism has not been reported.

In this research, the effects of PAW on the texture properties, rheological properties, microstructure, and chemical forces of DMP gel were studied. Then, the degree of MP aggregation, molecular weight distribution, level of oxidation, and conformational changes of DMP after PAW treatment were explored.

## 2. Materials and Methods

### 2.1. Materials

Ten 60-day-old male Cherry Valley ducks were purchased at a local butcher shop in Ningbo, Zhejiang Province, China, and humanely slaughtered, collecting muscles all over their bodies immediately after slaughter. All analytical grade chemicals were purchased from Macklin (Shanghai Macklin Biochemical Co., Shanghai, China).

### 2.2. Extraction of Myofibrillar Proteins

The extraction of DMP was conducted according to the method of Han et al. [20]. The fresh duck meat was minced in a meat grinder (JR07 Meat Grinder, Zhejiang Supor Co., Zhejiang, China), which was added to four volumes (*w*/*v*) of extraction buffer (100 mM Tris, 10 mM EDTA, pH = 8.3), homogenized in an ice bath for 30 s by a homogenizer (XHF-D High-Speed Dispersator, Ningbo Scientz Biotechnology Co., Ningbo, China), followed by centrifuging at 7000× *g* for 20 min. The pellet was redissolved in four volumes (*w*/*v*) of SSS solution (100 mM KCl, 20 mM Na_2_HCO_3_/NaH_2_CO_3_, 2 mM MgCl_2_, 1 mM EGTA, pH 7.0), followed by centrifugation at 10,000× *g* for 10 min. The pellet was redissolved in 1% Triton X-100 + 99% SSS, and the solution was centrifuged at 10,000× *g* for 10 min. After that, the pellet was washed twice with SSS. 100 mM KCl solution was added to the pellet, and the suspension was filtered with three layers of gauze to remove impurities. After centrifugation, 100 mM NaCl solution was added to the pellet. The precipitate was washed twice with water, and the resulting precipitate was MP after centrifugation. The concentration of MP obtained was determined by the Biuret method, and the protein was stored in a refrigerator at 4 °C and used within 24 h.

### 2.3. Preparation of PAW

The plasma-generating instrument used in this experiment was designed by Nanjing Prospect Electronics Technology Co. (Nanjing, China). The device consists of a plasma generator, a rotating nozzle and an air compressor. The carrier gas was air, and the airflow and power were set to 28–32 L/min and 680–700 W, respectively. The plasma jet nozzle was placed approximately 4 cm above 50 mL of liquid (20 mM phosphate buffer solution containing 0.6 M NaCl, pH = 7.2), and the liquid was stirred at 500 rpm and exposed to the plasma jet for 0–40 s (namely, control, PAW-10 s, PAW-20 s, PAW-30 s, and PAW-40 s), respectively. The protein was then diluted to 50 mg/mL by taking an appropriate amount of the generated PAW and incubated at 4 °C for 12 h before use.

### 2.4. Preparation of DMP Gels

The DMP gel was prepared according to the method of Shi et al. [21] with slight modifications. The PAW-treated DMP solution was poured into a 10 mL glass beaker, and the solution was heated from 25 °C to 45 °C at a rate of 2 °C/min for 30 min and then to 75 °C for 30 min. The heated samples were immediately cooled on ice for 30 min and then stored at 4 °C overnight for further testing.

### 2.5. Gel Strength

According to the method of Chen et al., [22], gel strength was analyzed at room temperature using a TA-XT2i texture analyzer (Stable Micro System Co., Godalming, UK). Tests were performed using a P/5 model probe under the following conditions: pre-speed, 1 mm/s; test speed, 2 mm/s; post-speed, 1 mm/s; distance, 10.0 mm; and trigger force, 5 g. The gel strength (g*mm) was expressed as the product of the breaking distance (mm) and breaking force (g). 

### 2.6. Water Holding Capacity

The water holding capacity (WHC) was measured according to the method of Wang et al. [23]. A total of 2 g of the gel was weighed in a centrifuge tube and centrifuged at 10,277× g for 10 min at 4 °C, and the supernatant was discarded. WHC was calculated as the ratio of the weight of the sample before and after centrifugation.

### 2.7. Dynamic Rheological Behavior

The rheological properties of MP were measured using Model DHR-2 rheometer (TA Instrument Co., New Castle, USA) according to the method of Li et al. [24]. The sample was placed between parallel plates of 40 mm diameter matched to the instrument. Before testing, the edges were sealed with liquid paraffin, and the sample was heated from 25 °C to 75 °C at a rate of 2 °C/min. The parameters were set as follows: constant frequency, 0.1 Hz; strain, 1%; gap, 1 mm. The storage modulus (G′) and loss modulus (G″) of the gelling process were recorded.

### 2.8. Molecular Forces

The chemical forces of the gels were determined according to the method of Gomez-Guillen et al. [25] by treating the gels with the following solutions: SA (0.05 M NaCl), SB (0.6 M NaCl), SC (0.6 M NaCl, 1.5 M urea) and SD (0.6 M NaCl, 8 M urea). one gram of chopped gel was homogenized in 9 mL of solution at a maximum rate for 2 min and left to stand for 1 h at 4 °C. The samples were then centrifuged at 10,277× *g* for 15 min at 4 °C, and the protein content of the supernatant was determined by the Biuret method. The protein concentration in SA represented the contribution of non-specific association, the difference in protein concentration between SB and SA solution represented the contribution of ionic bond, the difference of protein concentration between SC and SB solution represented the contribution of hydrogen bond, and the difference of protein concentration between SD and SC solution represented the contribution of the hydrophobic bond.

### 2.9. Microstructure

The gels were fixed in 2.5% glutaraldehyde for 12 h and then vacuumed and freeze-dried by a freeze-dryer (SCIENTZ-70FY Freeze-Dryer, Ningbo Scientz Biotechnology Co., Ningbo, China) according to the method of Jiang et al. [26]. The microstructure of the gels was observed using a scanning electron microscope (SEM) (Hitachi S-3400, Tokyo, Japan).

### 2.10. Protein Solubility

The solubility of myogenic fibrin was determined according to the method of Kong and Xiong [27] by diluting the MP solution to 2 mg/mL and then centrifuging at 5000× *g* for 15 min at 4 °C. The protein concentration was determined before and after centrifugation by the Biuret method, where protein solubility was defined as the ratio of the protein concentration in the supernatant to the protein concentration in the original suspension.

### 2.11. Gel Electrophoresis

According to the method of Xu et al. [28], 12% separating gel and 5% stacking gel were prepared with sodium dodecyl sulfate-polyacrylamide gel electrophoresis (SDS-PAGE) preparation kit (Sanggon Biotechnic Co., Shanghai, China). MP was dissolved in protein loading buffer and heated at 100 °C for 10 min using a digital dry bath (JX100-2, Shanghai Jingxin Experimental Technology Co., Shanghai, China) and then cooled to room temperature. Ten microliters of the sample was added to each swimming lane, and the protein marker (Solarbio, Beijing, China) with molecular weights of 11–245 kDa was added for comparative analysis. The electrophoresis was run at a voltage of 80 V in stacking gel and 120 V in separating gel. After electrophoresis, the samples were stained with Coomassie blue R-250. Images of the gels were collected using Gel DocTM XR + imaging system (Bio-Rad Laboratories Inc., Hercules, CA, USA).

### 2.12. Total Sulfhydryl Content

The concentration of the MP solution was adjusted to 5 mg/mL, and the total sulfhydryl content was determined according to the method of Ellman [29]. The total sulfhydryl content was calculated as a molar extinction coefficient of 13,600 m^−1^ cm^−1^, and the results were expressed as nmol SH/mg protein.

### 2.13. Carbonyl Content

The carbonyl content was determined according to the method of Zhang et al. [30] by diluting the MP solution to 5 mg/mL. The carbonyl content was calculated as a molar extinction coefficient of 22.0 m M^−1^ cm^−1^, and the results were expressed as nmol/mg protein.

### 2.14. Secondary Structure Analysis

Circular dichroism (CD) spectroscopy was performed at room temperature using the J-1500 spectropolarimeter (Jasco Co., Tokyo, Japan). The MP solution was diluted to 0.2 mg/mL, and 200 μL of the sample was added to a 0.1 cm cuvette. The scan rate, response time, and bandwidth were 100 nm min^−1^, 0.25 s, and 1 nm. The percentage of secondary structure was calculated using the instrument’s own protein secondary structure estimation program.

### 2.15. Surface Hydrophobicity

The surface hydrophobicity of the samples was determined according to the method of Chen et al. [31] by adding 1 mL of 1 mg/mL bromophenol blue (BPB) to 4 mL of MP solution, mixing, and reacting at room temperature for 15 min. The solution was then centrifuged at 8000× *g* for 15 min, the supernatant was diluted 10 times, and the absorbance at 595 nm was measured. Phosphate buffer solution and bromophenol blue solution were used as blanks.

### 2.16. UV Absorption Spectral Analysis

The UV absorption spectra of the samples were scanned in the range of 240–340 nm at room temperature using a UV-Vis spectrophotometer (UV-330, Shanghai Mapada Instruments Co., Shanghai, China) according to the method of Ekezie et al. [16]. 

### 2.17. Fluorescence Measurement

The MP solution was diluted to 0.2 mg/mL, and the fluorescence spectra were measured using a fluorescence spectrophotometer (F-4700, Hitachi, Japan) according to the method of Li et al. [32]. The excitation wavelength was 280 nm, the scanning wavelength was 300–500 nm, and the slit width was 5 nm. All samples shall be measured in triplicate.

### 2.18. Statistical Analysis

All of the experiments were performed in triplicate. Data were analyzed with One-way analysis of variance (ANOVA) using SPSS 26.0 software (IBM, Chicago, IL, USA) and compared differences between data using Duncan’s multiple range tests. The significance level was set to *p* < 0.05. Results were expressed as mean ± standard deviation. The required chart was drawn using Origin 2021 software (Origin Lab, Hampton, NH, USA).

## 3. Results and Discussion

### 3.1. Gel Strength and WHC

**Figure 1:**
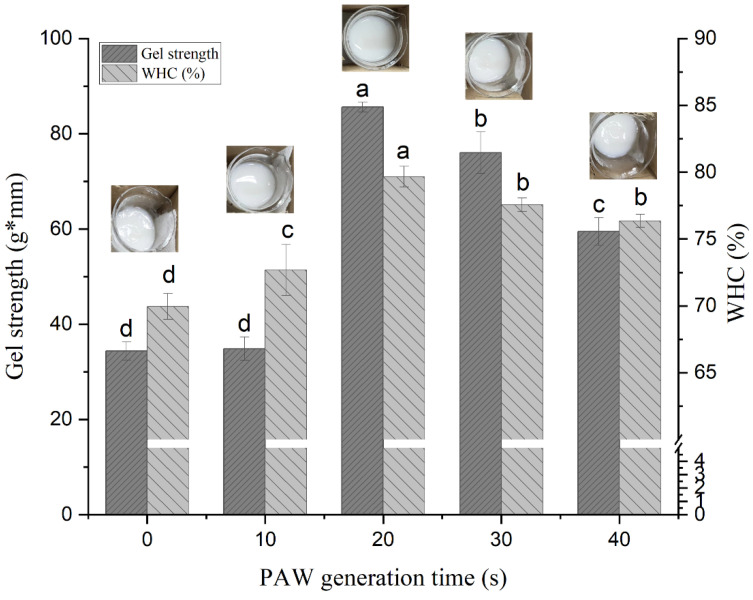
Effect of plasma-activated water on the gel strength and WHC of duck myofibrillar protein gel. Different letters indicate a significant difference between the groups.

Figure 1 shows that the gel strength of DMP gel varies with PAW generation time. With the increase of time, the gel strength first increased and then decreased, which is consistent with previous research results [33,34]. As the PAW generation time increased from 0 s to 20 s, the gel strength significantly improved about 2.5 times, from 34.37 ± 1.89 g·mm to 85.60 ± 1.06 g·mm. However, there was no significant difference between the gel strength in PAW-10 s group and the control group. PAW acquires specific properties by containing a variety of chemically reactive species with different half-lives, and these active species can undergo redox reactions with protein molecules to modify their structure [19]. During the early stages of the protein’s reaction with PAW, unfolded protein chains can be refolded due to the lower degree of oxidation at this time [35]. The gel strength showed a decreasing trend due to the higher degree of oxidation at the PAW-30 s and PAW-40 s, resulting in an impairment of gelling capacities [35]. This suggests that moderate plasma treatment improves the performance of the duck protein gel, while prolonged treatment deteriorates its performance.

The level of WHC depends on the ability of the spatial structure of the protein gel to retain water, which can reflect the stability of the gel structure [36,37]. PAW treatment leads to a certain degree of unfolding of DMP, which promotes the interaction between protein and water molecules, thereby affecting the water-holding capacity of the gel [38]. As shown in Figure 1, the WHC showed the same trend as gel strength, increasing from 69.95 ± 0.96% to 79.67 ± 0.77% as the PAW generation time increased from 0 s to 20 s and then decreased to 76.36 ± 0.49% at 40 s. The gel pictures in Figure 1 visually exhibited the status of the water overflow after forming the gel in different PAW treatment groups. The gels of PAW-20 s, PAW-30 s and PAW-40 s groups showed less water on the top, which also indicated a better WHC of the PAW gel system compared to the control. These findings were in agreement with those obtained by Sun et al. [39] and Wang et al. [40], who observed that low to moderate oxidation modification promoted the formation of a uniform and dense gel structure, while excessive oxidation resulted in a decrease in the WHC of the gel.

### 3.2. Dynamic Rheology

**Figure 2:**
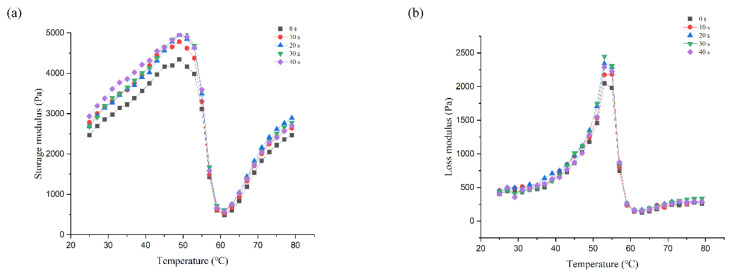
Effect of plasma-activated water on the rheological properties of duck myofibrillar protein during heating. (**a**) storage modulus G′; (**b**) loss modulus G″.

The storage modulus (G′) reflects the elastic changes of the PAW-treated MP solution throughout the heating process, which is affected by the intermolecular interaction of MP [41]. As shown in Figure 2a, G′ began to increase at 24 °C until it reached a maximum of 49 °C when the S1 subfragment began to unfold and denature, and myosin head-head interactions began to form gel networks [42]. Subsequently, G′ started to decrease at 49–61 °C, which was mainly due to the rearrangement of intermolecular and intramolecular forces caused by the denaturation of light meromyosin (LMM). The dissociation of the actin-myosin complex in this temperature range also causes a decrease in the storage modulus [43]. G′ increased sharply in the range of 61–79 °C, which is related to a permanent, irreversible gel network formation [44]. Throughout the heating process, the PAW-incubated DMP solution showed a larger storage modulus than the control group (Figure 2a), which may be due to the PAW treatment caused unfolding of MP structure and enhanced MP intermolecular crosslinking, therefore exhibiting higher elastic gelation during heating [45]. At the first developmental stage of G′ (25–49 °C), PAW-40 s showed a higher storage modulus than PAW-20 s and PAW-30 s, which may be attributed to the effects of hydrophobic interactions and disulfide bond formation [46]. However, in the second developmental stage (61–79 °C), PAW-20 s and PAW-30 s observed higher modulus of storage than PAW-40 s due to the inhibition of myosin head-head interactions while •OH promotes tail-tail interactions [47]. Ultimately, PAW-20 s and PAW-30 s form a more resilient gel network, exhibiting higher gel strength and WHC.

The loss modulus (G″) reflects the viscous behavior of the liquid-like components [48]. As shown in Figure 2b, similar to the G′ curve, the DMP with and without PAW treatment exhibited a typical G″ curve, peaking at around 50 °C. The initial and final G″ values of DMP with PAW added were not significantly different from the control group, but DMP treated with PAW showed higher peaks. In all experimental groups, PAW-20 s and PAW-30 s showed the maximum G″, which was similar to the result of G′. The results showed that the PAW-treated DMP had more elastic and viscous behavior compared to the control group, which facilitated the development of viscoelasticity in gels.

### 3.3. Molecular Forces

**Figure 3:**
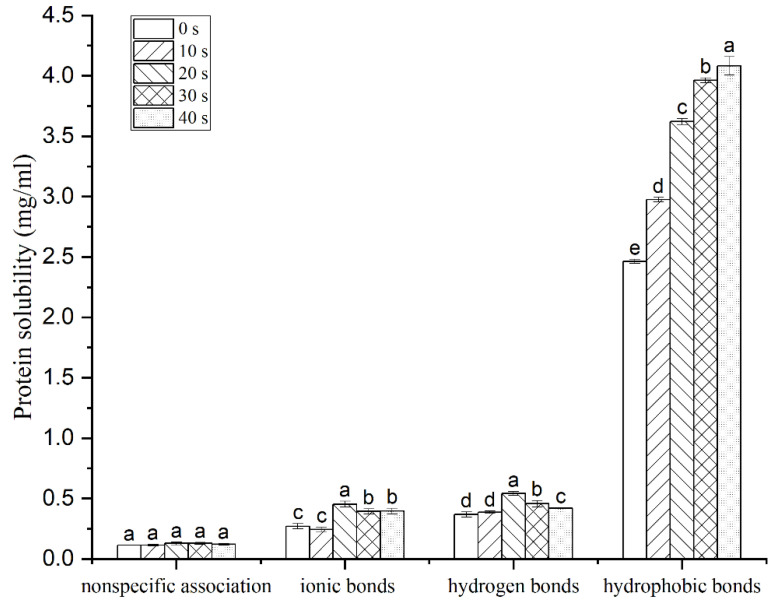
Effect of plasma-activated water on intermolecular forces in duck myofibrillar protein gel. Different letters indicate a significant difference between the groups.

The oxidative nature of PAW increases the degree of DMP denaturation and changes the structure, which inevitably affects the intermolecular interactions [49]. As shown in Figure 3, the content of nonspecific association, ionic bonds, and hydrogen bonds were significantly lower than the content of hydrophobic bonds, indicating that hydrophobic interaction was the main chemical force promoting gel formation, which was consistent with the results obtained by Mi et al. [50]. There was no significant difference in the nonspecific association content of DMP gel among different PAW treatment groups. Ionic bonds and hydrogen bonds are not involved in gel formation, but they can indirectly affect gelation properties by influencing the stability of protein conformation [51]. After the disruption of the ionic bond, the solubility of the protein increased from 0.27 ± 0.02 mg/mL (control) to 0.45 ± 0.02 mg/mL (PAW-20 s), which was 1.7 times that of the control group, however. As the PAW generation time increased to 40 s, the solubility decreased to 0.40 ± 0.02 mg/mL. The results of ionic bonds are inconsistent with those obtained by Li et al. [24], which may be attributed to differences in the raw materials and processes studied. The hydrogen bonds between MP and water molecules are the main force that captures free water in the gel network; therefore, the amount of hydrogen bonds is proportional to the level of WHC [52]. After PAW-20 s incubation, the hydrogen bond content was 1.5 times that of the control group, but the hydrogen bond content was significantly reduced after the PAW generation time was extended to 40 s. The change in hydrogen bond content was consistent with that of WHC. The content of hydrophobic bonds raised with the increase of PAW generation time and was 1.7 times that of the control group at 40 s, indicating that PAW treatment led to the exposure of hydrophobic groups of proteins, which was conducive to the formation of protein-protein aggregates and promoted the formation of a denser gel network [53,54]. However, excessive hydrophobic interactions produce typical Modori phenomena that cause the deterioration of gel structure [25].

### 3.4. The Microstructure

**Figure 4:**
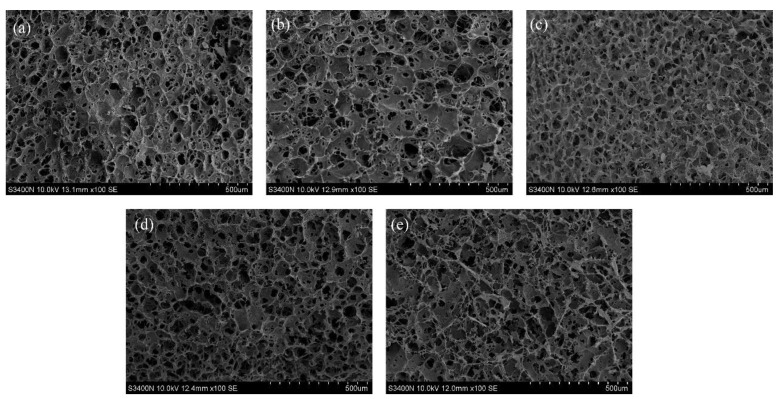
Effect of plasma-activated water on the microstructure of duck myofibrillar protein gels. (**a**–**e**) indicate the plasma-activated water generation time of 0 s,10 s,20 s,30 s, and 40 s, respectively.

Figure 4 shows the microstructure of gels with or without PAW. MP gels without PAW treatment exhibited an irregular and disordered spatial structure with some large holes on the surface (Figure 4a). In contrast, PAW-treated MP gels exhibited a more continuous, uniform, and tight, three-dimensional network structure (Figure 4c,d). The microstructure of the gel was more compact and orderly in PAW-20 s and PAW-30 s groups compared to other treatment groups, indicating that PAW generated under this time range promoted protein crosslinking in the gel. When the PAW generation time was increased to 40 s, it was observed that the gel network structure became coarse, which may be due to the destruction of the gel network structure due to excessive oxidation of MP induced by PAW. 

The gel network is facilitated by intermolecular covalent and non-covalent interactions, mainly hydrophobic interactions [55]. The addition of PAW enhances the hydrophobic interaction between MP molecules, forming a more orderly and tighter network. The dense gel structure leads to an increase in the contact area between proteins and water molecules filled in the gel voids, and the interaction between them leads to a decrease in water mobility, which explains why the PAW-treated gels have the higher water-holding capacity and better texture properties [56].

### 3.5. Solubility

**Figure 5:**
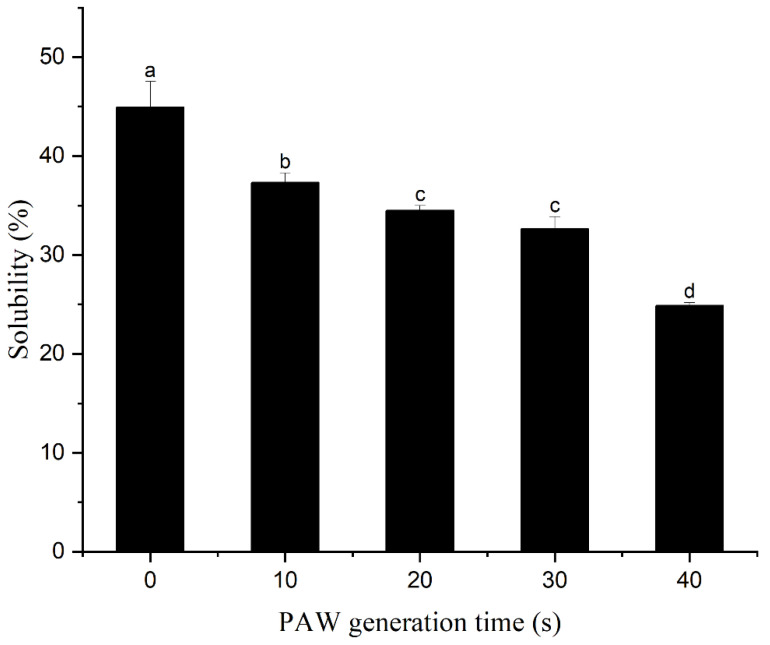
Effect of plasma-activated water on the solubility of duck myofibrillar protein. Different letters indicate a significant difference between the groups.

The solubility of protein is a good indicator of its degree of aggregation, which affects the functional properties of the protein [57]. As shown by Figure 5, with the increase of PAW generation time, the solubility of the protein gradually decreased, and by 40 s, the solubility had decreased to 24.88 ± 0.31%, which was 44.64% lower than that of the control group. It is noteworthy that the solubility trend is decreasing with the increase in PAW generation time, unlike other properties such as rheology and gel properties. This decrease in solubility is primarily attributed to the alteration of the protein’s structure. The PAW treatment modifies the tertiary structure of the protein, exposing the internal hydrophobic groups, which then interact with each other and cause protein aggregation, leading to a reduction in solubility [16]. Panpipat and Chaijan [58] showed the same result, where solubility decreased rapidly after treatment of fish MP with atmospheric pressure cold plasma and remained stable thereafter.

### 3.6. Electrophoresis Patterns

**Figure 6:**
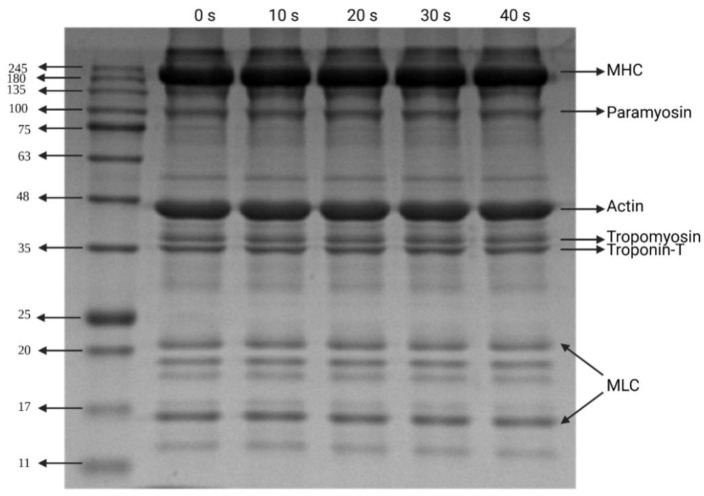
Electrophoresis pattern of duck myofibrillar protein treated with plasma-activated water as a function of plasma-activated water generation time (MHC, myosin heavy chain; MLC, myosin light chain).

Given the changes in protein patterns after PAW treatment, the samples were analyzed by SDS-PAGE. As shown in Figure 6, even with extended processing time, all samples presented similar band distribution, following the myosin heavy chain (220 kDa), paramyosin (100 kDa), actin (48 kDa), tropomyosin (36 kDa), troponin-T (35 kDa) and myosin light chain (11–25 kDa) [59]. The disappearance of bands and no extra fragment of dimerization was not observed with the naked eye, indicating that PAW treatment did not significantly alter the molecular weight of DMP or lead to the degradation of DMP under the process conditions adopted in this study. The aggregation phenomenon of MP after PAW treatment was not visualized in the electropherogram, indicating that covalent bonding was not the main cause of MP aggregation in duck meat. On the contrary, non-covalent interaction may be the main reason for promoting aggregation, and the results of intermolecular forces (Figure 3) also support this conclusion. Since SDS disrupts almost all non-covalent interactions in proteins, the effect of PAW on MP non-covalent interactions in duck meat is not visualized in electrophoresis [60]. The electrophoresis patterns obtained in this study suggested that the change of the primary structure of DMP induced by PAW was not predominant, which was also in agreement with the results obtained by Ekezie et al. [16], which showed that atmospheric pressure plasma jets did not change the protein pattern of shrimp MP. 

### 3.7. Sulfhydryl and Carbonyl Content

Sulfhydryl and carbonyl groups are indicators of protein oxidation and are used to characterize the degree of oxidation of duck MP after PAW treatment. As shown in Table 1, the sulfhydryl content of MP increased from 44.58 ± 0.92 nmol/mg to the highest value of 47.12 ± 0.71 nmol/mg with the PAW generated after 20 s, but the content decreased to 43.08 ± 1.54 nmol/mg in the PAW-40 s group. The change of sulfhydryl content is consistent with the finding of Sharifian et al. [15], in which the sulfhydryl content tends to increase in the early stages of PAW treatment due to the stretching and unfolding of protein molecules, where sulfhydryl groups originally buried in inaccessible sites inside the polypeptide chains are exposed to react with Ellman’s reagent. However, cysteine and methionine are very sensitive to ROS, and the sulfhydryl groups they contain are easily oxidized by ROS to produce intermolecular and intramolecular disulfide bonds and even further oxidized to sulfonic acid; therefore, the increase in sulfhydryl content is temporary [17]. Miao et al. [61] suggested that the decrease in sulfhydryl content is related to aggregation and crosslinking of protein molecules, and the exposed sulfhydryl groups are re-wrapped by aggregates.

Direct oxidation of susceptible amino acid side chains in proteins is the main pathway for protein carbonylation. Therefore, carbonyl content is often used as an indicator of the degree of protein oxidation [62]. From Table 1, it could be seen that the carbonyl content of the control group was about 1.14 ± 0.05 nmol/mg, and after 10–40 s generated PAW treatments, the content was 1.26, 1.56, 1.82, and 2.14 times that of the control group, respectively, indicating the deepening of protein oxidation. Threonine, proline and lysine in meat are easily oxidized to produce γ-glutamic semialdehyde (GGS) and α-amino adipic semialdehyde (AAS), which are the main carbonyl compounds for protein oxidation, accounting for more than 20% of the total carbonyl compounds [63]. The carbonyl fraction of AAS residues react with other AAS residues or lysine ε-amino groups to form aldol condensation structures and Schiff bases, and the formation of these intermolecular and intramolecular crosslinks is the main cause of changes in protein function [64]. It has also been suggested that mild to moderate oxidation of MP can increase the stability of the gel by forming crosslinks through carbonyl–amine condensations, while the random aggregation phenomenon caused by high oxidation can lead to loss of protein function [65].

### 3.8. Secondary Structure Changes of MP

**Figure 7:**
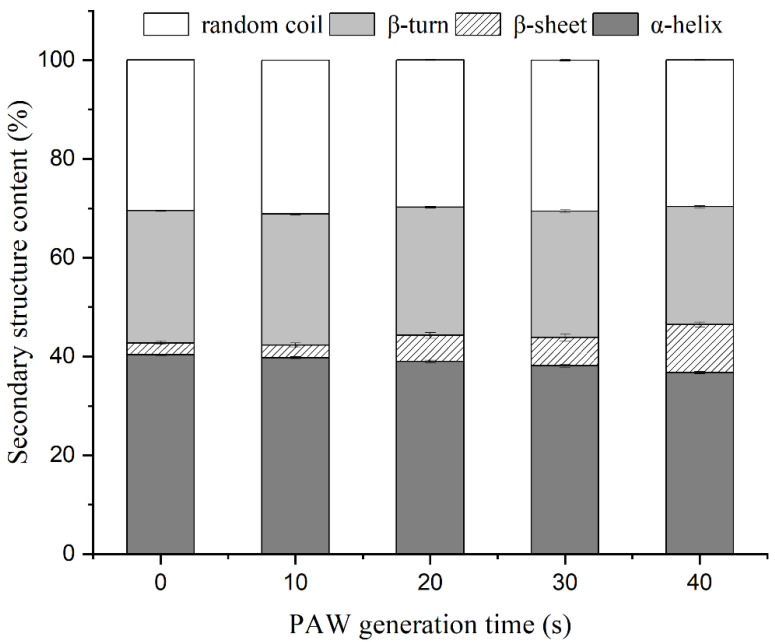
Effects of plasma-activated water on the secondary structure content of duck myofibrillar protein.

CD spectra of untreated and PAW-treated MP were used to obtain changes in the folding level of secondary structures. As shown in Figure 7, the secondary structure of MP was dominated by α-helix, and with the extension of PAW generation time, the content of α-helix gradually decreased from 40.33 ± 0.15% to 36.77 ± 0.25%, and the content of β-turn gradually decreased from 26.80 ± 0.10% to 23.90 ± 0.20%. However, the β-sheet content increased significantly from 2.40 ± 0.30% to 9.67 ± 0.50%. The results indicated that PAW treatment could result in a transformation of α-helix and β-turn into β-sheet in MP, which aligns with the findings obtained by Li et al. [24]. The increase in MP gel strength is strongly associated with the β-sheet content, and the water hydration strength of the β-sheet is weaker than that of the α-helix, which facilitates the formation of gel network structure [66]. This conclusion was also supported by the results of the study of Guo et al. [67], who observed that the gel strength of fish MP gel was inversely proportional to the α-helix content and directly proportional to the β-sheet content. Sharifian et al. [15] treated beef MP using dielectric barrier discharge plasma and observed a significant decrease in α-helix and β-turn structures within 10 min of treatment, which was attributed to protein structure opening.

### 3.9. Tertiary Structure of MP

**Figure 8:**
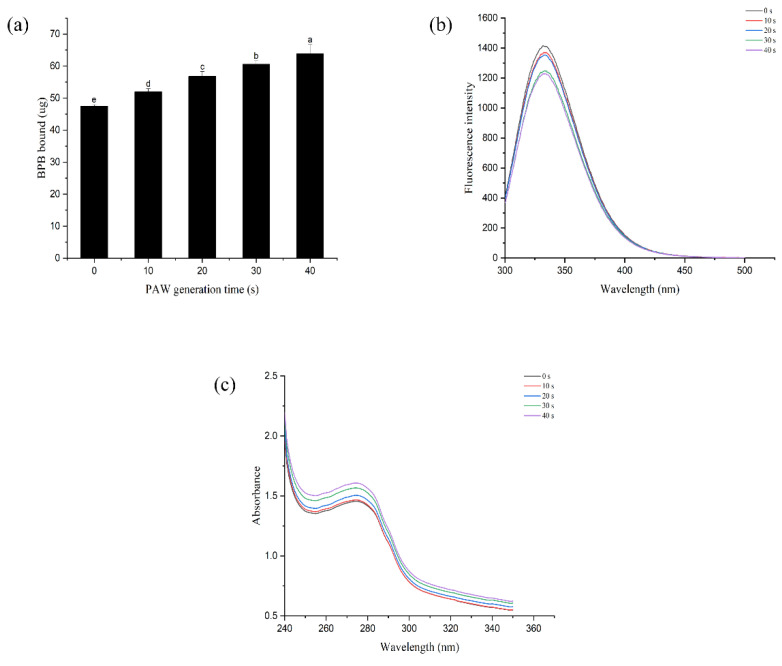
Effect of plasma-activated water on the tertiary structure of duck myofibrillar protein. (**a**) surface hydrophobicity, (**b**) fluorescence spectroscopy, (**c**) UV absorption spectroscopy. Different letters indicate a significant difference between the groups.

Surface hydrophobicity, fluorescence spectroscopy, and UV absorption spectroscopy reflect changes in the tertiary structure of proteins [68,69,70]. Changes in protein surface hydrophobicity are related to the number and distribution of hydrophobic groups on the protein surface, which are generally buried inside proteins and exposed to the surface as protein structures unfold [71]. As illustrated in Figure 8a, the surface hydrophobicity of DMP increased with the extension of PAW generation time, reaching 1.35 times that of the control group after 40 s. Similar results were also reported in previous studies [72]. The results suggested that PAW can alter the spatial structure of DMP, leading to more exposure to non-polar amino acids, which may be related to the ROS contained in PAW. Fu et al. [73] explored the effect of in vitro oxidation on the spatial structure of MP and observed that the surface hydrophobicity of MP was proportional to the concentration of hydrogen peroxide, which is one of the major reactive oxygen species contained in PAW. PAW treatment leads to the exposure of more hydrophobic groups, and these groups interact to cause protein aggregation, which ultimately results in a decrease in protein solubility [74].

The intrinsic fluorescence of MP can reflect changes in the microenvironment of protein fluorophores (mainly tryptophan residues) [75]. Typically, when MP is folded, tryptophan residues are located in a non-polar environment inside the protein (hydrophobic environment), exhibiting high fluorescence intensity. When the protein is partially or fully unfolded, tryptophan residues come into contact with solvents and are exposed to polar environments (hydrophilic environments), resulting in fluorescence quenching [76]. As shown in Figure 8b, samples without PAW treatment exhibited the highest fluorescence intensity. The addition of PAW caused the fluorescence quenching of DMP, and the maximal emission wavelength was also shifted from 331.8 nm red to 333.8 nm. A similar result has been observed in the case of pea protein [77]. The above results showed the change of tryptophan residues from a hydrophobic to a hydrophilic environment. Zhu et al. [78] also observed that the fluorescence intensity of MP in fish decreased with increasing hydrogen peroxide concentration. PAW treatment induces DMP unfolding, exposing hydrophobic amino acids hidden inside, and the results of UV absorption spectroscopy also supported this conclusion. As shown by Figure 8c, protein absorption at 273 nm is ascribed to the combined effects of tyrosine, tryptophan, and phenylalanine [79]. The absorption intensity of the PAW-treated sample increased significantly with the increase in PAW generation time, indicating an increased trend of protein aggregation. This means that MP reacts with active substances contained in PAW and exposes more hydrophobic groups, causing enhanced cross-linking between proteins [45].

## 4. Conclusions

In conclusion, the use of PAW with a short generation time significantly improved the quality of DMP gels. This could be a result of the active species in the PAW gently oxidizing DMP and altering its spatial structure, which exposes hydrophobic groups and increases the potential for cross-linking between protein molecules, ultimately creating favorable conditions for protein aggregation. The results of this study highlight that the PAW generated by short-term CP treatment can enhance protein flexibility and improve functional properties. Conversely, strong oxidation effects caused by prolonged PAW generation can lead to the destruction of the gel structure. PAW is a promising tool for developing new products in the food industry, but in practical applications, the process control of PAW production should be considered to inhibit the excessive oxidation of proteins. Furthermore, due to the diversity of active species contained in plasma-activated water, a more comprehensive mechanism by which PAW promotes DMP gelation needs to be understood.

## Figures and Tables

**Table 1:** Changes in sulfhydryl content and carbonyl content of duck myofibrillar protein after plasma-activated water treatment.

PAW Generation Time (s)	0	10	20	30	40
Sulfhydryl groups (nmol/mg protein)	44.58 ± 0.92 ^bc^	45.73 ± 0.18 ^ab^	47.12 ± 0.71 ^a^	45.17 ± 0.91 ^b^	43.08 ± 1.54 ^c^
Carbonyl content (nmol/mg protein)	1.14 ± 0.05 ^e^	1.44 ± 0.06 ^d^	1.78 ± 0.02 ^c^	2.07 ± 0.06 ^b^	2.44 ± 0.14 ^a^

Results were expressed as means ± standard deviations (n = 3). Different letters indicate a significant difference between the groups.

## Data Availability

The data are avaliable from the corresponding author.
